# Effects of different protocols of hydration on cardiorespiratory parameters during exercise and recovery

**DOI:** 10.1186/1755-7682-6-33

**Published:** 2013-08-23

**Authors:** Franciele Marques Vanderlei, Isadora Lessa Moreno, Luiz Carlos Marques Vanderlei, Carlos Marcelo Pastre, Luiz Carlos de Abreu, Celso Ferreira

**Affiliations:** 1 Doctoral student in Medicine (Cardiology), Federal University of São Paulo – UNIFESP, São Paulo, SP, Brazil; 2 Department of Physiotherapy, Paulista State University – FCT/UNESP, Presidente Prudente, SP, Brazil; 3 Department of Physiotherapy, ABC Faculty of Medicine – FMABC, São Paulo, SP, Brazil; 4 Department of Medicine (Cardiology), Federal University of São Paulo – UNIFESP, Rua Napoleão de Barros, 715 - Térreo, São Paulo, SP, Brazil

**Keywords:** Aerobic exercise, Rehydration solutions, Heart rate, Blood pressure, Respiratory rate

## Abstract

**Introduction:**

Hydration plays a key role in the physiological maintenance required by exercise.

**Objective:**

To evaluate the behavior of heart rate (HR), systolic (SBP) and diastolic (DBP) blood pressure, pulse oxygen saturation (SpO_2_) and respiratory rate (RR) of young people during and after prolonged physical exercise, with and without the intake of water or isotonic solution.

**Method:**

31 young individuals (21.63 ± 1.86 years) were subjected to a four-step protocol with a 48-hour interval between each step, namely: i) a test to determine the incremental load used in subsequent steps, ii) a control protocol without hydration (CP), iii) an experimental protocol with water intake (PE1), iv) an experimental protocol with ingestion of isotonic (PE2). The protocols consisted of 10 min rest, 90 min of exercise on a treadmill at 60% of VO_2_peak and 60 min of recovery. The parameters HR, SBP, DBP, RR and SPO_2_ were measured at rest, at 30, 60 and 90 min of exercise, with the exception of RR; and at 1, 3, 5, 7, 10, 20, 30, 40, 50 and 60 min of recovery. The two-factor analysis of variance for repeated measures model was used for analysis (p<0.05).

**Results:**

There was a moment effect for all variables in exercise (p<0.001), however, no effect was observed between the protocols (SBP, p=0.998; DBP, p=0.897; SpO_2_, p=0.077, HR=0.281) and in the interaction moment and protocol (SBP, p=0.058; DBP, p=0.191 and SpO_2_, p=0.510, HR=0.496). In recovery there was also a moment effect for all variables analyzed (p<0.001). There was no effect among protocols for SBP (p=0.986), DBP (p=0.536) and RR (p=0.539), however in the SpO_2_ (p=0.001) and HR (p=0.033) variables, effects were observed between the protocols. Regarding the moment and protocol interaction, an effect was observed for HR (SBP, p=0.431; DBP, p=0.086; SpO_2_, p=0.445, RR, p=0.147, HR, p=0.022).

**Conclusion:**

For the type of exercise performed, both the water and the isotonic solution influenced the behavior of cardiorespiratory parameters, and independent of the type of hydration given the behavior of the parameters studied was similar.

## Introduction

Dehydration caused by prolonged exercise limits the ability to work [1,2] and promotes, among other things, inadequate responses of the cardiovascular, autonomic and thermoregulation systems [3-6].

Different studies have shown that hydration plays an essential role in the tolerance of prolonged exercise, suggesting that it promotes a better response to increased body temperature [2,7] and ensures the maintenance of physiological functions and the proper functioning of homeostatic mechanisms required by the practice of exercise [8,9], helping to maintain blood pressure and cardiac output constant, thus supporting the increase in cutaneous blood flow and sweat rate regulation according to the body temperature [3].

Hydration can be achieved in several ways, among them water [10], isotonic solution [11], pasteurized milk [12], infusion of pure glucose [13] and solution of pure sodium chloride [14]. Currently, researchers in the field of thermoregulation strive to detect possible advantages and determine cost / benefit, especially in the case of hydration with water and isotonic solution.

Drinking water provides rapid gastric emptying without the need to adjust palatability and offers a low financial cost [8], whereas the intake of carbohydrates and electrolytes present in isotonic solution brings additional benefits to the ability to perform, whether by increasing energy availability, or aiding in fluid balance and temperature regulation [11].

Considering the organic changes promoted by exercise-induced dehydration, it is important to study the changes that may occur during and after prolonged exercise when water or isotonic solution is used as a source of replacement. A search of the literature showed no relevant studies that evaluate and compare the effects of these two forms of hydration on cardiorespiratory parameters when administered equally during exercise and throughout the recovery period. It is hypothesized that regardless of the rehydration solution administered, during prolonged exercise there is a lower overload of the cardiorespiratory parameters and better recovery of these parameters after its completion.

Thus, the objective of this study was to evaluate the behavior of the heart rate (HR), systolic (SBP) and diastolic (DBP) blood pressure, pulse oxygen saturation (SpO_2_) and respiratory rate (RR) of young people during and after prolonged physical exercise, with and without the intake of water or isotonic solution.

## Method

### Population

To conduct this study, we analyzed data from 31 apparently healthy, male volunteers, with a mean age of 21.63 ± 1.86 years, and classified as active by the International Physical Activity Questionnaire (IPAQ) [15].

Excluded form the study were volunteers who had at least one of the following characteristics: smoking, use of medications that would influence the autonomic activity of the heart, alcoholics, patients with known metabolic and/or endocrine disorders, or a sedentary, or insufficiently active or overactive lifestyle according to IPAQ. During the execution of the experimental protocol not a single volunteer was excluded.

The volunteers were informed of the objectives and procedures of this study, and after agreeing, signed a consent form. All procedures were approved by the Ethics Committee of the Federal University of São Paulo via Platform Brazil (CA0AE: 02481012.0.1001.5505) and conformed with resolution 196/96 of the National Health Council.

### Study design

All volunteers were subjected to an experimental procedure divided into four stages, with an interval of 48 hours between each stage to allow for recovery. The first step performed was an incremental test on a treadmill to determine peak oxygen consumption (peak VO_2_) and to adopt 60% of this value for the load used in the other stages.

In the subsequent steps the volunteers performed three different protocols, namely the control protocol (CP), the hydration with water protocol (PE1) and the hydration with isotonic drink protocol (PE2), which consisted of 10 min of rest in the supine position, followed by 90 min of exercise (60% VO_2_peak) and 60 min of recovery. In CP no hydrating solution was administered, whereas in PE1 water was given, and PE2 received a hydroelectrolytic solution (Gatorade, Brazil): the liquids were administered in 10 equal portions at regular 15-minute intervals, commencing 15 minutes after the start of exercising until the end of the recovery period.

Before performing these steps, the volunteers were subjected to a urine collection, for analysis of the degree of hydration, and body weight and height were measured. The body temperature was measured at the end of the rest period prior to exercise and at the end of the exercise period. For the assessment of the cardiorespiratory parameters SBP, DBP, HR, SpO_2_ and RR were measured. All these parameters were measured at the end of the 10th minute of rest pre-exercise and during exercise at 30, 60 and 90 min, except for RR which was not evaluated in the exercise. In the recovery period all parameters were evaluated at 1, 3, 5, 7, 10, 20, 30, 40, 50 and 60 min. The volunteers’ body weight was measured again at the end of the recovery period.

### Incremental test

The incremental exercise test was performed on a treadmill (Super ATL, Inbrasport, Brazil), according to the maximal Bruce protocol [16], to determine peak oxygen consumption (peak VO_2_) and adopt 60% of this value for the load used in the following steps. During this procedure, the subjects were at rest on the mat in the standing position in order to stabilize the initial values of the cardiorespiratory parameters, and then the test started. To determine oxygen consumption (VO_2_), expired gases were analyzed by a regularly calibrated metabolic analyzer (VO2000, Medical Graphics, St. Paul, MN, USA) [10]. The VO_2_peak was considered the highest VO_2_ achieved in the test, and verbal encouragement was used in an attempt to obtain a maximum physical effort, and the test was interrupted by voluntary exhaustion.

### Control and experimental protocols

All protocols were performed in a room with an average temperature of 26.0°C ± 2.34 and humidity of 55.13 ± 10.40 %, between the hours of 15 and 18 to avoid circadian variation. In order to ensure initial hydration condition, subjects ingested 500 ml of water, two hours before the protocols [17]. To perform all the steps, the volunteers were instructed to refrain from consuming caffeine-based drinks for 24 hours before the tests and to have a light, fruit-based meal, two hours before, to avoid intense physical activities the day before the test and to be dressed in appropriate and comfortable clothing (shorts, shirt, shoes and socks).

For each protocol performed, upon arrival at the collection site the following were measured: the volunteers’ weight, standing undressed (Plenna digital scale, TIN 00,139 MAXIMA, Brazil) and height (stadiometer ES 2020 - Sanny, Brazil). An HR (Polar Electro - S810i, Kempele, Finland) heart monitor was strapped over the region of the precordium and the receiver attached to the wrist to record this parameter and the volunteers were asked to rest in the supine position for 10 minutes. Immediately after, the axillary temperature (thermometer BD Thermofácil, China), HR, SBP, DBP, RR and SpO_2_ were measured.

Then, the subjects performed 90 minutes of treadmill exercise (60% VO_2_peak) and at 30, 60 and 90 min their HR, SBP, DBP, and SpO_2_ were measured. At the end of the exercise, the subjects were asked to rest in the supine position, to begin 60 min of recovery, and the axillary temperature was again recorded. The HR, SBP, DBP, SpO_2_ and RR parameters were measured at 1, 3, 5, 7, 10, 20, 30, 40, 50 and 60 min of recovery. At the end of that time, the undressed subjects’ weight was measured again.

The amount of water or isotonic fluid administered during exercise and recovery in PE1 and PE2 was based on the difference in body weight measured before and after CP. This technique implies that the loss of one gram of body mass is equivalent to a milliliter of lost fluid [18]. Furthermore, in PE1 and PE2, the measurement of the cardiorespiratory parameters in the coinciding moments occurred immediately after ingestion of the respective solutions.

### Analysis of cardiorespiratory parameters

The analysis of HR was undertaken by means of a cardiac frequency counter (Polar Electro Oy - S810i model), equipment previously validated to capture beat-to-beat HR [19,20]. A blood pressure measurement was taken indirectly using a stethoscope (Littmann, Saint Paul, USA) and an aneroid sphygmomanometer (Welch Allyn - Tycos, New York, USA) on the left arm of volunteers, according to the criteria established by VI^th^ Brazilian Guidelines on Hypertension [21]. RR measures were performed by counting breaths for 1 min without the volunteer being aware of the process, so that the usual characteristics of breathing were not modified. SpO_2_ was verified by a pulse oximeter (Mindray PM-50 Pulse Oximeter, China).

### Statistical analysis

For the data analysis of the population’s profile the descriptive statistical method was used and the results were presented as mean values, standard deviations, median, minimum and maximum values. Data normality was assessed using the Shapiro-Wills test.

For weight and body temperature, the comparison between the moments of the same protocol was performed using the Student t test for unpaired data when the distribution was normal or the Wilcoxon test for data with non-normal distribution. For analysis between protocols, one-way ANOVA was used with post Tukey test for normally distributed data or the Kruskal Wallis with Dunn’s post test for non-normal data.

The comparisons of cardiorespiratory parameters between protocols (CP vs. PE1 vs. PE2) and moments (rest vs. time in exercise and rest vs. recovery time) was made by means of the variance analysis technique for repeated measures model in the two-factors scheme. The measurement data were checked for repeated violation of sphericity using Mauchly’s test and the Greenhouse-Geisser correction was used when sphericity was violated.

For analysis of the moments (rest vs. time in exercise and rest vs. recovery time) the Bonferroni post-test for parametric distribution was used or the Dunnet post-test for nonparametric distribution, and analysis of different moments between groups was accomplished using One-Way ANOVA or the Kruskal Wallis test. Statistical significance was set at 5% for all analyzes, and calculations were performed using SPSS software - version 13.0 (SPSS Inc., Chicago, IL, USA).

## Results

Table 1 shows the anthropometric characteristics of the study population jointly with the results obtained in the incremental test.

**Table 1:** **Mean values**, **followed by the standard deviation**, **and median of anthropometric variables and the incremental test of analyzed individuals**

**Variables**	**Mean ± standard deviation (Median)**	**Minimum - Maximum**
Age (years)	21.63 ± 1.86 (22.00)	18.00 – 25.00
Weight (Kg)	72.62 ± 11.53 (72.20)	53.80 – 95.30
Height (m)	1.77 ± 0.08 (1.78)	1.60 – 1.94
BMI (Kg.m^2^)	22.99 ± 2.83 (22.30)	16.87 – 28.07
VO_2pico_ (l.min^-1^)	3.37 ± 0.60 (3.34)	2.02 – 5.14
60% VO_2pico_ (l.min^-1^)	2.02 ± 0.36 (2.00)	1.21 – 3.08
HR (bpm)	160.74 ± 10.75 (162.00)	139.00 – 179.00

**Legend**: *BMI* body mass index, *kg* kilograms, *m* meters, *VO*_*2*_*peak* peak oxygen consumption; *l* liter, *min* minute, *HR* heart rate, *bpm* beats per minute.

As can be seen in Table 2, the values of initial temperature and weight in CP differ statistically with respect to final values, with reduced weight and increased temperature. In PE1 and PE2 only a significant increase in body temperature was observed. Comparing the protocols, only the final body temperature of CP showed higher values compared to PE2 (p <0.05).

**Table 2:** **Mean values**, **followed by the standard deviation and median**, **the variables weight and body temperature of individuals analyzed in the CP**, **PE1 and PE2**

**Variables**	**Time**	**CP**	**PE1**	**PE2**
**Weight (Kg)**	**Initial**	73.03 ± 11.56* (72.90)	73.05 ± 11.40 (73.20)	72.90 ± 11.50 (72.20)
	**Final**	71.55 ± 11.30 (71.00)	72.97 ± 11.52 (72.60)	73.08 ±11.51 (72.60)
**Body Temperature (°C)**	**Initial**	36.44 ± 0.47# (36.50)	36.45 ± 0.35* (36.50)	36.30 ± 0.37# (36.50)
	**Final**	37.20 ± 0.53† (37.20)	37.04 ± 0.45 (37.00)	36.83 ± 0.42 (36.80)

*Value statistically compared with the end (Student’s t test for paired data, p <0.05); # Value with statistical difference compared to the end (Wilcoxon test, p <0.05); † Value with statistical difference in relation to PE2 (ANOVA with Tukey posttest, p <0.05).

**Legend**: *kg* kilogram; *°C* degrees Celsius; *CP* Control Protocol; *PE1* Experimental Protocol with water ingestion; *PE2* Experimental Protocol with isotonic intake.

The analysis showed that during exercise there was a moment effect for all variables analyzed (p <0.001). In this condition no effect was observed between the protocols (SBP, p = 0.998; DBP, p = 0.897; SpO_2_, p = 0.077, HR = 0.281) and interaction moment and protocol (SBP, p = 0.058; DBP, p = 0.191 and SpO_2_, p = 0.510, HR = 0.496).

**Figure 1:**
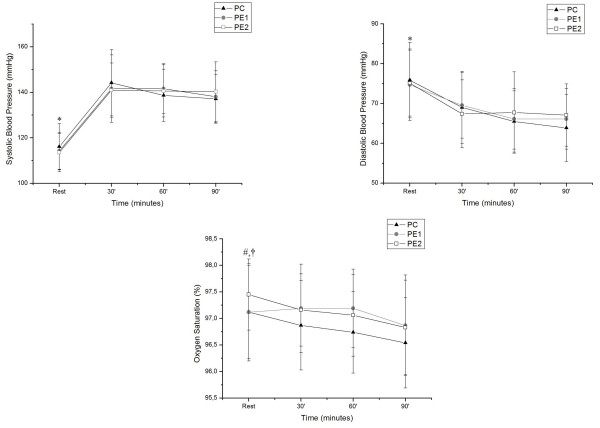
**Behavior of SBP, DBP and SpO**_**2 **_**during exercise and their comparison in relation to the initial resting in PC, PE1 and PE2.** Legend: Mean values, followed by the standard deviation of SBP, DBP and SpO_2_ obtained from the CP, PE1 and PE2 at rest and during exercise; * Value with statistical difference in the 30, 60 and 90 min. in CP, PE1 and PE2 (p <0.05); # Amount statistical difference compared with the 90 min. CP (p <0.05); † Value Statistically compared to 60 and 90 min in PE2 (p <0.05). CP = Control Protocol; PE1 = Experimental Protocol with water ingestion; PE2 = Experimental Protocol with isotonic intake.

The results for the SBP, DBP and SpO_2_ variables at rest and during exercise are shown in Figure 1 in all protocols significant differences were observed in the SBP and DBP variables when rest is compared with 30, 60 and 90 min of exercise, with the same occurring with HR. For SpO_2_ a significant decrease was observed at the 90 min mark compared with rest in CP, while in PE2, there was a significant decrease at the times of 60 and 90 min compared to rest.

In recovery, the moment effect was observed for all variables (p <0.001). There was no effect between protocols for SBP (p = 0.986), DBP (p = 0.536) and RR (p = 0.539), however for the SpO_2_ (p = 0.001) and HR (p = 0.033) variables, effects were observed between the protocols. Regarding the interaction moment and protocol, an effect was observed only for HR (SBP, p = 0.431; DBP, p = 0.086; SpO_2_, p = 0.445; RR, p = 0.147; HR, p = 0.022).

**Figure 2:**
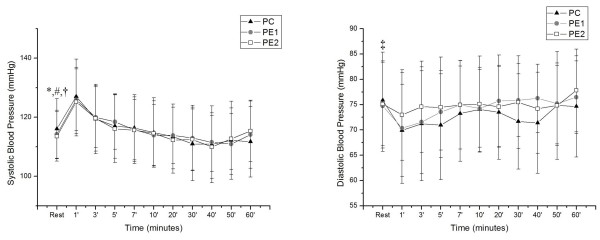
**Behavior of SBP and DBP during recovery and their comparison in relation to the initial resting in PC, PE1 and PE2.** Legend: Mean values, followed by the standard deviations of the variables SBP and DBP obtained from CP, PE1 and PE2 at rest and during recovery; * Value with statistical difference compared to 1, 30, 40, 50 and 60 min. CP (p <0.05); # Amount statistical difference compared with the 1, 3 and 5 min. in PE1 (p <0.05); † Value with statistical difference compared to 1 and 3 min. in PE2 (p <0.05); ‡ Value with statistical difference compared to the 1, 3, 5, 30 and 40 min. CP (p <0.05). CP = Control Protocol; PE1 = Experimental Protocol with water ingestion; PE2 = Experimental Protocol with isotonic intake.

Significant differences were observed for SBP in CP between rest and at 1, 30, 40, 50 and 60 min of recovery. For PE1, rest differed at 1, 3 and 5 min, and in PE2 at 1 and 3 min. As for DBP, only in CP did rest show significant differences compared to times 1, 3, 5, 30 and 40 min (Figure 2).

**Figure 3:**
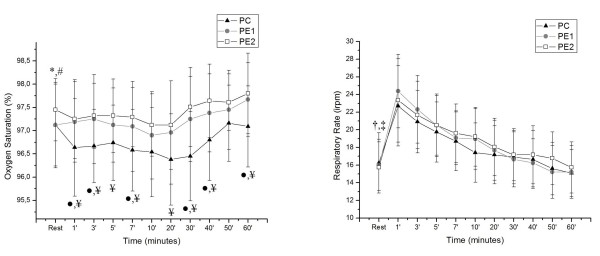
**Behavior of RR and SpO**_**2 **_**during recovery and their comparison in relation to the initial resting in PC, PE1 and PE2.** Legend: Mean values, followed by the standard deviations of the variables obtained from RR and SpO_2_ from CP, PE1 and PE2 at rest and during recovery; * Value with statistical difference compared to 7, 10, 20 and 30 min. CP (p <0.05); # Amount statistical difference compared with the 60 min. in PE1 (p <0.05); † Value with statistical difference compared to the 1, 3, 5 and 7 min. CP (p <0.05); ‡ Value with statistical difference compared to the 1, 3, 5, 7, 10 and 20 min. in PE1 and PE2 (p <0.05); • Value with statistical difference between CP and PE1; ¥ Value with statistical difference between the CP and PE2 (p <0.05). CP = Control Protocol; PE1 = Experimental Protocol with water ingestion; PE2 = Experimental Protocol with isotonic intake.

The results obtained for RR and SpO_2_ at rest and during recovery are shown in Figure 3. For the SpO_2_ variable in CP, the values at 7, 10, 20 and 30 min were lower compared to rest, while in PE1, rest differed only at 60 min, showing a slight increase. In the comparison between the protocols except at the 50 min mark, lower values of SpO_2_ were observed for CP compared to PE2, and the same occurred in the comparison between CP and PE1 at 1, 3, 7, 30, 40 and 60 min. As for RR in CP, rest differed from 1, 3, 5 and 7 min, while in PE1 and PE2 rest differed from 1, 3, 5, 7, 10 and 20 min.

**Figure 4:**
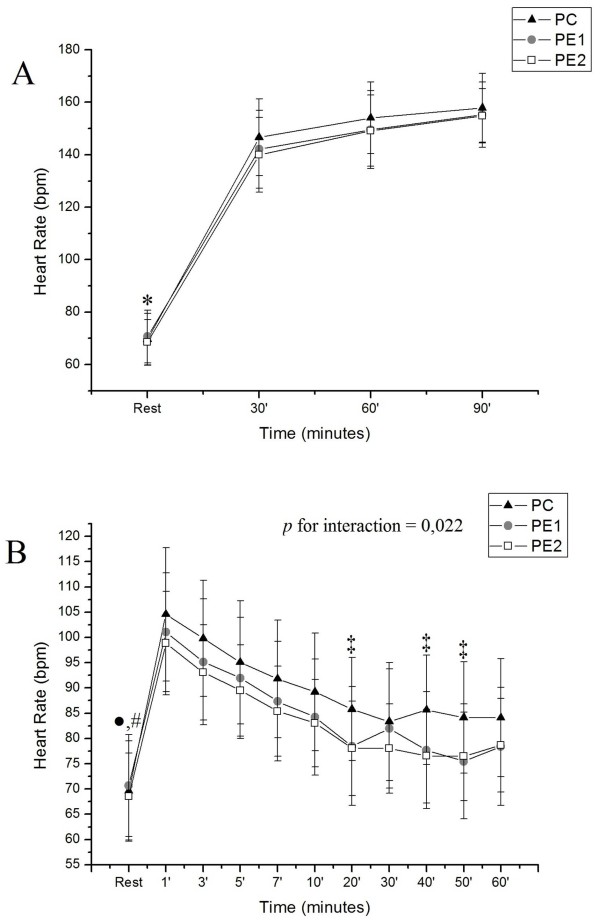
**Behavior of RH during exercise (A) and recovery (B) and their comparison in relation to the initial resting in PC, PE1 and PE2.** Legend: Mean values, followed by the standard deviation of the variable obtained in HR from CP, PE1 and PE2 at rest and during exercise **(A)** and recovery **(B)**. * Value with statistical difference in the 30, 60 and 90 min. CP, PE1 and PE2 (p <0.05); • Value with statistical difference in relation to all times on CP and PE2 (p <0.05); # Value with statistical difference compared to the 1, 3, 5, 7, 10 and 30 min. in PE1 (p <0.05); ‡ Statistically Value CP compared aoPE1 and PE2 (p <0.05). CP = Control Protocol; PE1 = Experimental Protocol with water ingestion; PE2 = Experimental Protocol with isotonic intake.

Figure 4 shows the behavior of HR in the three protocols conducted during exercise (A) and recovery (B). In CP and PE2, rest was statistically different from all other stages of recovery, while in PE1, rest differed from times 1, 3, 5, 7, 10 and 30 min. In the comparison between protocols, the values obtained in CP were higher compared with PE1 and PE2 at 20, 40 and 50 min.

## Discussion

The present study evaluated in young individuals the influence of hydration carried out with water or isotonic solution on the cardiopulmonary parameters during and after prolonged, submaximal exercise. The results showed that both hydration with isotonic solution and with water influenced the behavior of cardiorespiratory parameters, promoting minor fluctuations in these variables during the execution of the exercise and more efficient recovery, with a faster return to baseline levels of these parameters. Furthermore, data indicated that for the type of exercise performed, regardless of the type of hydration given, the behavior of cardiorespiratory parameters studied was similar.

During exercise there was an increase in SBP and a reduction in DBP compared to rest. In general, during exercise, SBP increases in proportion to the increase in cardiac output [22], while DBP can reduce or maintain its values as a result of higher blood absorption by muscle capillaries [4], so the blood pressure responses observed in this study are normal and expected for the type of exercise performed.

During exercise, there were reductions in SBP and DBP between the times of 30 and 90 min, with the greatest decreases in CP. Reductions in SBP and DBP were also observed by other authors after two hours of exercise in hypohydrated condition, which did not occur when hydration was maintained [23]. Significant reductions in SBP and mean blood pressure following an hour of exercise in dehydrated condition with maintenance of DBP were also reported in the literature [24].

The greatest reductions observed in CP may be related, at least in part, to the reduced stroke volume caused by the decrease in blood volume and venous return that occurred with these volunteers due to the quantity of liquid lost. Body mass reduction after performing exercises indicates fluid loss [25], which was observed in CP volunteers but was absent in the protocols in which volunteers received hydration. Another aspect that may be involved with the greater reduction in the blood pressure values in CP was the higher rise in body temperature that was observed in this protocol, which is associated with vasodilatation and consequently reduced blood pressure [26].

The decrease in SpO_2_ observed during the performance of physical exercise in the three protocols may be considered physiological and expected. The analyzed volunteers were healthy and without respiratory distress, and during moderate or intense physical activity, the blood flows more quickly through the alveolar capillaries, and consequently there is less time for exchanges between the hemoglobin and the alveolar air, which reduces the SpO_2_[27]. Moreover, the working muscles need a steady increase in the supply of oxygen and the inability of the circulatory system to provide this linear distribution, may also be related to the decrease in SpO_2_[28].

As for HR, the highest values observed during exercise in the CP can be explained by the decrease in plasma volume that occurs with hypohydration. This condition decreases the systolic volume and in compensation, HR increases in an attempt to maintain cardiac output and hence, blood flow to the active muscles in order to meet the metabolic needs, and to the skin, enabling the body’s temperature reduction mechanism through perspiration. [29]. Montain and Coyle (1992) [30] demonstrated that the increase in body temperature and heart rate, as well as the decrease in systolic volume during exercise are directly related to the degree of dehydration of the individual.

Lower HR values during the execution of prolonged exercise among individuals hydrated with water [13] or isotonic solution [31] were reported. When hydration occurs during exercise, it appears to reduce the increase in HR and the decrease in stroke volume [25]. Callegaro et al. (2007) [32] observed that up to 35 minutes after the ingestion of 500 ml water, blood pressure and vascular resistance increased and HR decreased. The authors suggest that hydration leads to an increase in the sympathetic vasoconstrictor and thus triggers a blood pressure response that stimulates the reflex vagal modulation, decreasing HR.

In all protocols immediately after exercise (1 min) SBP values remained high in relation to rest, which occurs due to the body’s need to effectively coordinate the various metabolic responses, such as increased blood flow to the skeletal muscle and critical tissues such as the heart and brain [33].

The reduction in sympathetic activity and the increase in vagal tone which occurs in recovery lead to a decrease in HR and peripheral resistance, thereby decreasing cardiac output and, therefore, SBP [33], which can be seen in the protocols performed.

In CP, SBP at the end of the recovery period studied (60 minutes) showed lower values than the basal resting rate, which may be related to water loss associated with increased body temperature that occurs in this protocol, which promotes a greater drop in cardiac output and therefore a greater reduction in SBP [33].

DBP values did not change when comparing recovery times with rest in the protocols where there was fluid replacement. However, in CP, a significant drop in DBP in the first minutes of recovery was observed, which may be related to greater increases in body temperature demonstrated after exercise in this protocol. In order to assist the process of heat dissipation, the cardiovascular system reduces peripheral resistance, promoting vasodilation with concomitant reduction in DBP [7,34].

Hydration with water as well as with sports drinks, had slight effects on blood pressure, so the differences found throughout the recovery period may be considered casual. Brown et al. (2005) [35] evaluated the role of hydration carried out at rest with water and saline on the cardiovascular responses of healthy young men and found that hydration had little effect on blood pressure in the course of an hour following hydration. Moreno et al. (2012) [31], when analyzing submaximal prolonged exercise in active young individuals, also observed that the administration of electrolytic solution ingested during the entire period of exercise and recovery, promoted higher values of SBP in the early minutes of recovery compared to rest, while the values of DBP remained constant.

Although we did not observe interaction between moment and protocol, differences occurred between the groups and moments for SpO_2_ during the recovery period. According to González-Alonso and Calbet (2003) [36] after exercise, reduction in muscle blood flow and oxygen supply occur due to decreased cardiac output and mean blood pressure produced by the reduction in VO_2_max in response to heat. Thus, we can infer that the hydrated condition allowed the maintenance of SpO_2_ by protecting the circulatory system.

As for RR, in the first minutes of recovery higher values were observed when compared to rest. One hypothesis for this is that the physical and chemical stimuli produced by exercise, such as decreasing pH, increasing the temperature and elevation of catecholamines in the blood, promote an elevation of RR [37,38]. Furthermore, the increase in gas exchange in the alveoli which occurs during exercise promotes increased RR [37,38].

In general, HR decreased progressively during the recovery period in the three tests, however, there are differences between protocols and interaction between moment and protocol (p = 0.022), indicating that hydration, independent of the solution used, provided better HR recovery when compared to the condition without hydration.

The faster recovery of HR in the hydration protocols used can be related to two aspects. Some authors have shown that hydration provides increased baroreflex sensitivity, a sharp decrease in sympathetic activity and consequently increased cardiac parasympathetic modulation [39,40], which could be responsible for HR recovering faster. Furthermore, in the hypohydrated condition the increased body temperature stimulates a higher sinus node firing rate [41], which might also be implicated in the slower HR recovery which occurred in CP.

It is noteworthy that although the hydration protocols provided better HR recovery, a return to baseline values was not observed. Khanna and Manna (2005) [42], after the administration of 100 ml of hydroelectrolytic solution found that 20 min recovery were insufficient for the return of HR to pre-exercise values, while another study showed that the return to baseline conditions did not occur after 60 min of recovery with the ingestion of isotonic solution [31].

In general, except for some casual differences, regarding type, intensity and duration of the proposed exercise (submaximal aerobic, 60% of VO_2_max, 90 min), both hydration with water or isotonic solution promoted the same effects on cardiorespiratory parameters.

Much has been discussed about the need for electrolyte replacement during rehydration. According to some studies [2,43,44], dehydration above 2% of body weight may impair physiological function and influence physical performance, due to loss of water and electrolytes. Studying the impact of hydration with water or saline on physiological responses while performing aerobic exercise at 55% VO_2_max., for six hours, with a temperature of 30°C and relative humidity of 50%, Barr et al. (1991) [45] indicated that the replacement of sodium seems not to be necessary during moderate events lasting less than 6 hours.

Moreover, Wootton (1988) [46] reported that in several liters of sweat lost during exercise, the proportion of existing electrolytes is minimal and Katch and McArdle (1990) [47] reported that in one liter of sweat, sodium loss corresponds to a mere 1.5 g, which can easily be replaced by normal daily nutrition.

In the present study, we found that in CP the body weight loss was an average of 1.48 kg corresponding to 2.02% of body weight. This suggests that the loss of electrolytes produced by exercise may have been insufficient for the isotonic solution to improve the cardiorespiratory parameters more efficiently when compared to water, since replacement with either water or isotonic solution proved sufficient in stabilizing cardiorespiratory parameters in exercise and in recovery more efficiently.

Finally, the study limitations should be considered. The evaluations performed during rest and recovery were made in the supine position, as this is more tolerable and comfortable for the volunteers, while the exercise was performed in the standing position. However, we believe that changes in cardiorespiratory parameters observed in exercise compared to rest occur in the same way regardless of position, and comparisons between rest and recovery were performed in the same position, which validates the comparison between them.

## Conclusion

Both hydration with isotonic solution and with water influenced the behavior of cardiorespiratory parameters, promoting during the execution of the exercise, minor variations in these parameters and more efficient recovery, with faster return to basal conditions. Furthermore, data indicated that for the type of exercise performed, regardless of the type of hydration given, the behavior of cardiorespiratory parameters studied was similar.

## Abbreviations

HR: Heart rate; SBP: Systolic blood pressure; DBP: Diastolic blood pressure; SpO2: Pulse oxygen saturation; RR: respiratory rate; CP: Control protocol; PE1: Experimental protocol with water intake; PE2: Experimental protocol with ingestion of isotonic; IPAQ: International Physical Activity Questionnaire; VO2peak: Peak oxygen consumption; min: Minutes.

## Competing interests

The authors declare that they have no competing interests.

## Authors’ contributions

FMV, ILM, LCMV and CF conceived of the study, participated in its design and coordination and helped to draft the manuscript. FMV, ILM, LCMV, CMP, LCA and CF performed the statistical analysis and interpretation of data and prepared the draft manuscript. All authors participated in the design of the study and in critical review of the manuscript. All authors read and approved the final manuscript.
